# Pneumonia risk prediction in patients with acute alcohol withdrawal syndrome through evaluation of sarcopenia index as a prognostic factor

**DOI:** 10.1186/s12877-023-03792-7

**Published:** 2023-02-08

**Authors:** Lingdan Zhao, Sha Huang, Fu Jing, Ting-ting Yu, Zeng Wei, Xiaoyan Chen

**Affiliations:** 1 Zigong Affiliated Hospital of Southwest Medical University, Zigong Psychiatric Research Center, Zigong, Sichuan Province China; 2 School of Nursing, Southwest Medical University, Luzhou, Sichuan China

**Keywords:** Sarcopenia index, Alcohol withdrawal syndrome (AWS), Pneumonia, Serum creatinine, Serum cystatin-C, Risk

## Abstract

**Objective:**

This study aimed to explore the relationship between the sarcopenia index (SI) and the risk of pneumonia in hospitalized patients with acute alcohol withdrawal syndrome (AWS).

**Study design:**

We have performed a retrospective study of individuals with AWS from a teaching hospital in western China. Patients' data were retrieved from the medicinal record databases. Patients' primary (upon admission) blood serum creatinine (Cr) and cystatin C (CysC) levels were incorporated into the records. Participants were separated into low and high SI cohorts based on the three-quarter digit of SI (SI = serum Cr/serum CysC ratio × 100). The association between SI and the risk of pneumonia in hospitalized patients with AWS was assessed by logistic regression analysis.

**Result:**

Three hundred and twelve patients with acute AWS were included in this retrospective analysis. Among hospitalized patients with acute AWS, the incidence of pneumonia was 13.78%. The average median age of acute AWS patients with pneumonia was 55.28 (10.65) years, and the mean age of acute AWS individuals without pneumonia was 51.23 (10.08) years. In the univariate analysis, the high SI group (SI > 87.91) had a lower incidence of pneumonia than the low SI group (SI ≤ 87.91) (high SI vs. low SI, 6.41% vs. 16.24%, *p* = 0.029). Further logistic regression analysis showed that the high SI group demonstrated a poorer risk of pneumonia (OR = 0.353, 95%CI: 0.134–0.932, *p* = 0.036). After adjusting for possible confounders, the risk of pneumonia remained low in the high SI group (OR = 0.358, 95%CI: 0.132–0.968, *p* = 0.043).

**Conclusion:**

Our results showed that SI was linked with the risk of pneumonia in hospitalized individuals with acute AWS. We further suggest that it could be a pneumonia risk factor, especially in medical centers where sarcopenia diagnosis is unavailable.

## Introduction

AWS is a common clinical syndrome [1], classified as the most general and potentially disabling, life-threatening complication of unhealthy alcohol use [2, 3]. It is defined as a series of clinical manifestations, including tremors, agitation, nausea, sweating, vomiting, hallucinations, insomnia, tachycardia, hypertension, delirium, and seizures that occur when a patient with alcohol dependence abruptly stops drinking, whether intentionally or unintentionally [4]. It is estimated that pneumonia is the leading cause of hospitalizations in patients with AWS [5]. Among adult patients hospitalized for trauma, the rate of pneumonia in the AWS group was 12%, more than five times the rate in non-AWS patients [6]. In intensive care units, one-third of AWS patients developed pneumonia [7]. After pneumonia, AWS patients had a higher risk of hospitalization and intensive care unit admission than non-AWS patients [8]. Therefore, screening for the incidence of pneumonia is essential. Studies have demonstrated that sarcopenia is a significant risk factor for community-acquired pneumonia in the elderly, post-gastrectomy pneumonia in patients with gastric cancer, and pneumonia in patients with alcoholic hepatitis [9–11].

Sarcopenia is a progressive, widespread disorder of the skeletal muscles characterized by a rapid loss of muscle mass and function [12] associated with an increase in adverse outcomes (including falls, functional decline, frailty, pneumonia, and death) [9–12]. Current methods of accurately measuring muscle mass require specific instruments [13]. However, this is impossible due to a lack of equipment in some medical institutions, especially psychiatric hospitals. On the other hand, some patients with acute AWS have some difficulty completing diagnostic tests for sarcopenia. For example, some patients with acute AWS have tremors or mental symptoms [14], preventing them from completing the Dual-energy X-ray absorptiometry (DXA, or DEXA) and InBody examinations. In addition, to date, there is no recognized cut-off value for diagnosing sarcopenia by computed tomography (CT) and magnetic resonance imaging (MRI). Therefore, a more straightforward method to screen for sarcopenia in acute AWS patients is needed. Researchers propose the sarcopenia index (SI) as a surrogate screening index for sarcopenia [15–18]. The SI is calculated as a ratio between the blood serum concentrations of creatinine (Cr) and cystatin C (CysC).

However, there are no data regarding the correlation between SI and pneumonia in AWS patients. Therefore, this study aimed to assess the effectiveness of SI in predicting the risk of pneumonia in hospitalized patients with acute AWS.

## Methods

### Study design and characteristics of the individuals involved in the study

AWS patients treated at a psychiatric teaching hospital in western China from April 28, 2017, to June 1, 2021, were included in the retrospective study. Men with acute AWS (within seven days of cessation) were involved. Our study subjects were all hospitalized for acute AWS. Chronic alcohol consumption was defined as the average daily consumption of 50 g of white wine and continuous drinking for one year or more [19]. Only the first hospitalization data were included if the patient was hospitalized multiple times. Exclusion criteria include: 1) an estimated glomerular filtration rate (eGFR) less than 15 ml/min/1.73 m^2^; 2) the patient was not checked for serum Cr or CysC; 3) having a terminal stage of the malignant tumor.

### Ethics

The Center for Health Informatics anonymized all relevant data, and reviewed the study protocol for this retrospective medical records-based investigation. Data confidentiality was maintained at all times, and our research followed the guidelines of the Declaration of Helsinki. Being retrospective in nature, this research did not require informed patient consent. Finally, we received approval for this study from our Research Ethics Committee (No. 202209).

### Data collection

Patients' general and pneumonia data were extracted from the electronic medical record system. Available clinical data included age, height, weight, smoking history, drinking time, daily alcohol consumption, drinking index, high blood pressure, type 2 diabetes mellitus, coronary heart disease (CHD) and chronic obstructive pulmonary disease (COPD), arrhythmia, lacunar infarction, together with post-admission blood test results (albumin [ALB], eGFR). The method of drinking index was calculated by following the formula: daily drinking amount (g/day) * drinking years [19] (alcohol gradus: 52°). Patients with pneumonia were identified based on the diagnosis in the discharge certificate consistent with ICD-10. We referred to the methods of other published literature [7], did not distinguish between community-acquired pneumonia and hospital-acquired pneumonia, and we included pneumonia that occurred during hospitalization for acute AWS.

### Sarcopenia index (SI)

Starving venous blood was analyzed (after an 8-h overnight fast) by an experienced psychiatric nurse. First, the SI was calculated by the formula: serum Cr/CysC × 100 [17, 20]. Then, the individuals were split into groups based on the three-quarter-digit SI rates [21].

### Statistical analysis

The SPSS 25.0 software was used to evaluate all results. Normal distributed continuous results are expressed as mean ± standard deviation (SD); otherwise, they are expressed as the median and interquartile range (IQR). Categorical data were exemplified as amounts (%). In addition, patients' standard medical data were analyzed by the Student's t-test, Pearson's chi-square, Rank-sum test, and the logistic regression analysis to define the association between SI and pneumonia risk in patients with acute AWS. Our study has two models: the unadjusted model (Model 1) and Model 2, which adjusts for possible confounding variables. Due to the high value of the drinking index when it was used as a continuous variable, it was added to the model as a categorical variable to correct it. The classification was evenly split according to the three-quarters quantile so that less than or equal to the three-quarters quantile was the low group, and greater than the three-quarters quantile was the high group [21].

## Results

**Fig. 1:**
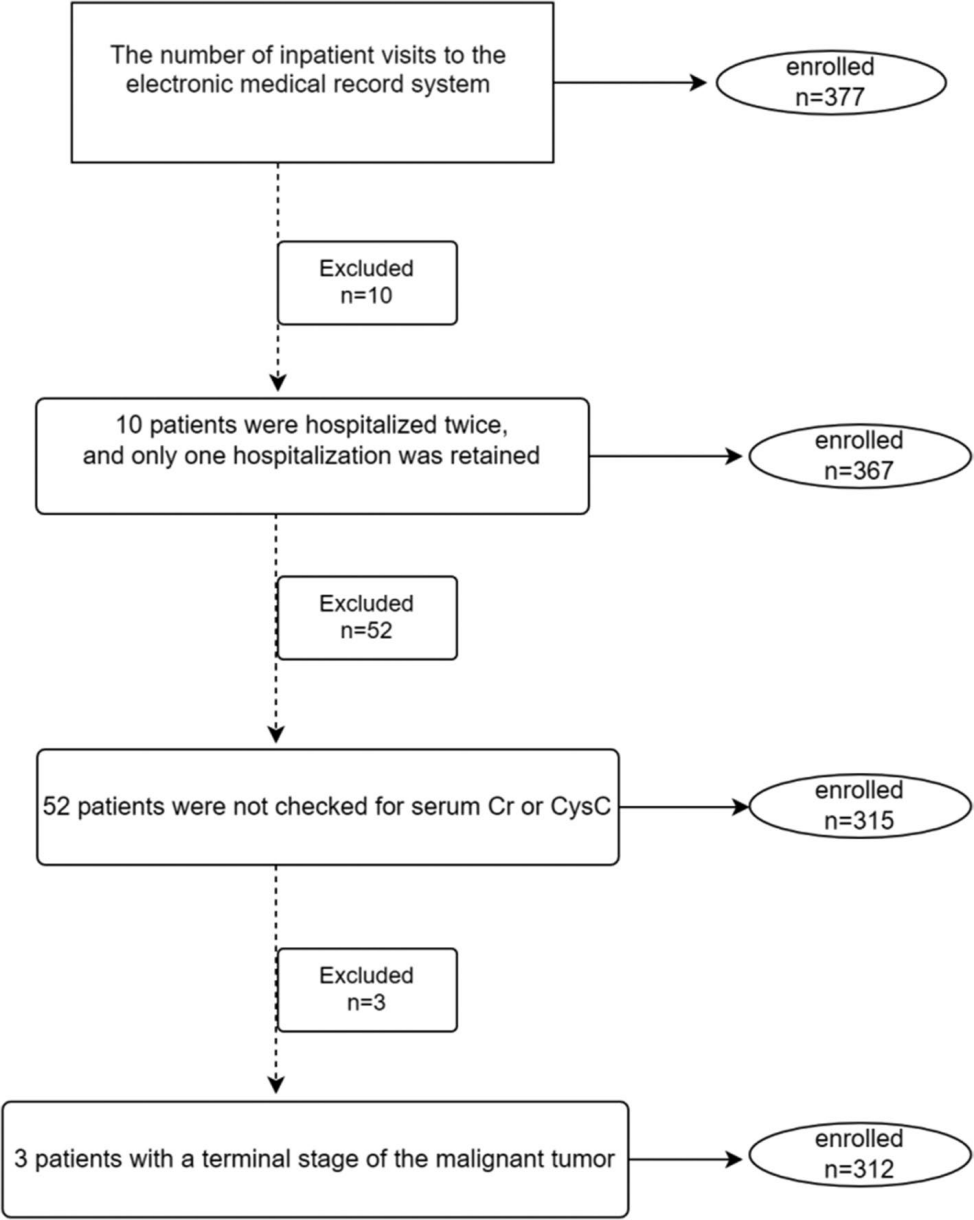
The study profile included patient selection information

The number of inpatient visits to the electronic medical record system was 377, from whom we included 312 patients with acute AWS and analyzed their data (Fig. 1). Among hospitalized patients with acute AWS, the incidence of pneumonia was 13.78%. The acute AWS patients included in the study developed pneumonia within 30 days of admission, and 42 patients developed pneumonia within 14 days of admission. The mean age of acute AWS patients with pneumonia was 55.28 (10.65) years, and the mean age of acute AWS patients without pneumonia was 51.23 (10.08) years. There were differences in the age, drinking index, and ALB levels between pneumonia and non-pneumonia patients (Table 1). However, the smoking history, duration of drinking, daily alcohol consumption, COPD, hypertension, diabetes, CHD, arrhythmia, lacunar infarction, body mass index (BMI), and eGFR in patients with and without pneumonia were not statistically substantial (Table 1).

**Table 1:** Characteristics of the study population

General characteristics	Non-pneumonia *n* = 269	Pneumonia *n* = 43	*P*
**Age, year, n (%)**			0.003
**< 60**	212(89.45)	25(10.55)	
**≥ 60**	57(76)	18(24)	
**Smoking history, n (%)**			0.404
no	16(80)	4(20)	
yes	253(86.64)	39(13.36)	
**Duration of drinking, year, median(iqr)**	30(20,32)	30(22,40)	0.05
**Alcohol consumption/day, g, median(iqr)**	400(250,500)	500(250,500)	0.051
**Drinking index, median(iqr)**	10,000(5575,15,000)	12,000(9000,18,000)	0.035*
**≤ 15,000, n (%)**	224(83.27)	45(16.73)	
**> 15,000, n (%)**	30(69.77)	13(30.23)	
**COPD, n (%)**			0.07
no	260(86.96)	39(13.04)	
yes	9(69.23)	4(30.77)	
**Hypertension, n (%)**			0.894
no	223(86.1)	36(13.9)	
yes	46(86.79)	7(13.21)	
**Diabetes, n (%)**			0.674
no	248(85.81)	41(14.19)	
yes	21(91.3)	2(8.7)	
**CHD, n (%)**			0.884
no	267(86.41)	42(13.59)	
yes	2(66.67)	1(33.33)	
**Arrhythmology, n (%)**			0.863
no	251(85.96)	41(14.04)	
yes	18(90)	2(10)	
**Lacunar infarction, n (%)**			0.343
no	244(86.83)	37(13.17)	
yes	25(80.65)	6(19.35)	
**BMI, kg/m**^**2**^**, median(iqr)**	20.22(18.46,22.15)	19.1(17.54,21.69)	0.099
**ALB, g/l, n (%)**			0.013
< 35	12(67.67)	6(33.33)	
≥ 35	257(87.41)	37(12.59)	
**eGFR, ml/min/1.73m**^**2**^**, median(iqr)**	120.4(99.97,144)	119.36(99.67,149.9)	0.88

*COPD* Chronic obstructive pulmonary disease, *CHD* Coronary heart disease, *BMI* Body mass index, *ALB* Albumin, *eGFR* Estimated glomerular filtration rate

^*^ As a continuous variable, the difference between the two groups was statistically significant (*P* < 0.05)

Individuals with SI scores of 87.91 and below were assigned to the low SI group, whereas the rest were allocated to the high SI group. There was a statistically significant difference in eGFR between the low SI group and the high SI group of acute AWS patients (Table 2). However, there were no statistically significant differences in age, smoking history, duration of drinking, alcohol consumption, drinking index, COPD, hypertension, diabetes, CHD, arrhythmology, lacunar infarction, BMI, and ALB (Table 2). The univariate analysis revealed that the high SI group had a lower incidence of pneumonia than the low SI group (6.41% vs. 16.24%, *p* = 0.029; Table 3). Further logistic regression analysis revealed that the high SI group had a lower risk of pneumonia than the low SI group (OR = 0.353, 95%CI: 0.134–0.932; Table 4). After adjusting for potential confounders, the high SI group continued to have a lower risk of developing pneumonia (OR = 0.358, 95%CI: 0.132–0.968; Table 4). Model 2 involved variables with *p* < 0.05 in univariate analysis and variables that may affect the accuracy of the results (COPD and diabetes). BMI was not included in the analysis model. In univariate analysis, there was no statistically significant difference between acute AWS patients with and without pneumonia. On the other hand, the relationship between BMI and the risk of pneumonia is complicated [22].

**Table 2:** Characteristics of the study population according to the sarcopenia index

General characteristics	Low SI *n* = 234	High SI *n* = 78	*P*
**Age, year, n (%)**			0.939
**< 60**	178(75.11)	59(24.89)	
**≥ 60**	56(74.67)	19(25.33)	
**Smoking history, n (%)**			0.109
no	12(60)	8(40)	
yes	222(76.03)	70(23.97)	
**Duration of drinking, year, median(iqr)**	30(20, 34.25)	27(20, 31.5)	0.115
**Alcohol consumption/day, g, median(iqr)**	400(250, 500)	400(268.75, 500)	0.609
**Drinking index, median(iqr)**	10,000(6000, 15,000)	9887.5(5000,15,000)	0.545^#^
**≤ 15,000,n(%)**	192(75.59)	62(24.41)	
**> 15,000, n (%)**	42(72.41)	16(27.59)	
**COPD, n (%)**			0.624
no	223(74.58)	76(25.42)	
yes	11(84.62)	2(15.38)	
**Hypertension, n (%)**			0.068
no	189(72.97)	70(27.03)	
yes	45(84.91)	8(15.09)	
**Diabetes, n (%)**			0.707
no	216(74.74)	73(25.26)	
yes	18(78.26)	5(21.74)	
**CHD, n (%)**			0.576
no	231(74.76)	78(25.24)	
yes	3(100)	0	
**Arrhythmology, n (%)**			0.79
no	218(74.66)	74(25.34)	
yes	16(80)	4(20)	
**Lacunar infarction, n (%)**			0.229
no	208(74.02)	73(25.98)	
yes	26(83.87)	5(16.13)	
**BMI, kg/m**^**2**^**, median(iqr)**	20.03(18.4, 22.05)	20.51(18.35, 22.3)	0.452
**ALB, g/l, n (%)**			0.262
< 35	16(88.89)	2(11.11)	
≥ 35	218(74.15)	76(25.85)	
**eGFR, ml/min/1.73m**^**2**^**, median(iqr)**	125.6(107.79, 150.89)	100.62(88.54, 121.03)	< 0.001

*COPD* chronic obstructive pulmonary disease, *CHD* Coronary heart disease, *BMI* Body mass index, *ALB* Albumin, *eGFR* Estimated glomerular filtration rate

^#^ *P* ≥ 0.05

**Table 3:** Univariate analysis of SI and pneumonia

Variable	Non-pneumonia *n* = 269	Pneumonia *n* = 43	*P*
SI			0.029
Low SI	196(83.76)	38(16.24)	
High SI	73(93.59)	5(6.41)	

*SI* Sarcopenia index. Low SI group: SI ≤ 87.91; high SI group: SI > 87.91

**Table 4:** Correlations between SI and pneumonia

Variable	Model 1		Model 2	
	***P*-value**	**OR (95% *CI*)**	***P*-value**	**OR (95% *CI*)**
SI				
Low SI	-	1	-	1
High SI	0.036	0.353(0.134–0.932)	0.043	0.358(0.132–0.968)

Model 1: a non-adjusted model

Model 2 (SI group): adjusting for age, drinking index, ALB level, COPD, diabetes

Low SI group: SI ≤ 87.91; high SI group: SI > 87.91

## Discussion

Our results proved the high SI disincentive for pneumonia in hospitalized patients with acute AWS. To the best of our knowledge, this is the first study to explore SI and pneumonia risk in hospitalized patients with acute AWS. These results further suggest that SI can be a risk predictor factor of pneumonia among these individuals, especially where the diagnosis of sarcopenia is unavailable.

In our study, the incidence of pneumonia in hospitalized patients with acute AWS was 13.78%, higher than that of the general population [23]. The following are the possible mechanisms: First, heavy alcohol consumption and sedative medications suppress the central nervous system and cough reflexes, resulting in impaired airway clearance. In addition, AWS patients may exhibit clinical symptoms such as vomiting, delirium, hallucinations, and epilepsy. Due to the inhalation of bacteria into the oropharynx, patients with AWS are more susceptible to pneumonia [4, 24, 25] Second, alcohol inhibits CXC chemokine production [26], S100 protein response [27], tumor necrosis factor-alpha, macrophage inflammatory protein-2 production, and recruitment of neutrophils and lymphocytes to the lung [28, 29]. In addition, ethanol inhibits CD11b/c activity on employed neutrophils and the phagocytic activity of blood-circulating neutrophils [28]. Finally, ethanol reduces splenocytes and affects spleen function [30].

We showed that AWS individuals in the low SI cohort displayed a high risk of developing pneumonia. In other words, sarcopenia was associated with pneumonia in the AWS population. Some authors showed that sarcopenia was closely linked to decreased swallowing and coughing function [31]. Additionally, sarcopenia is associated with impaired immune cell function, including neutrophils and lymphocytes [32, 33]. Muscle also produces and secrete cytokines, including IL-6, IL-7, and IL-15, which regulate the immune system [32, 33]. Therefore, the swallowing, coughing, and immune function of AWS patients in the low SI group appeared worse. Therefore, in clinical practice, we recommend that clinicians pay greater attention to sarcopenia-specific treatment, such as nutritional therapy and exercise therapy [13], in AWS patients assessed as low SI group. This might reduce the incidence of pneumonia in patients with AWS, thereby improving the adverse clinical outcome.

Our investigation had several limits, which we need to address. First, the number of patients was small, while the study was retrospective and observational, which probably led to possible selection bias. A more extensive cohort investigation is desirable to check the conclusions. This is because the larger cohorts provide data that allow reliable statistical evaluation, such as propensity score analysis or inverse probability weighting (IPW). Second, we did not include women in the analysis because of the small number of women (only 2) among the acute AWS patients screened in this study. Finally, the accuracy of SI as a screening indicator for sarcopenia might also be compromised when renal impairment is severe. Therefore, in many studies, patients with severe renal impairment were excluded [15, 17, 34]. Our study also used an eGFR of less than 15 ml/min/1.73 m^2^ as an exclusion criterion. We need to stress here that some medical institutions are unable to check Cr and CysC at the same time.

## Conclusion

Our results showed that SI is associated with pneumonia risk in hospitalized individuals with acute AWS. They further proved that it could be used as a prognostic factor of pneumonia risk factor in hospitalized patients with acute AWS in medical settings where sarcopenia diagnosis is unavailable.

## Data Availability

The datasets generated and analyzed during the current study are not publicly available due to this is a database which has a lot of important information and we are applying some important projects based on this. But this data sets will be available 2 years later and is also available now from the corresponding author on a reasonable request.
